# Adherence to HAART: Africans Take Medicines More Faithfully than North Americans

**DOI:** 10.1371/journal.pmed.0040083

**Published:** 2007-02-27

**Authors:** Amir Attaran

In 2001, the chief of the United States Agency for International Development (USAID), Andrew Natsios, gave this justification to the US Congress for why the agency opposed giving antiretroviral therapy (ART) to Africans with HIV:

“If we had [HIV medicines for Africa] today, we could not distribute them. We could not administer the program because we do not have the doctors, we do not have the roads, we do not have the cold chain…[Africans] do not know what watches and clocks are. They do not use western means for telling time. They use the sun. These drugs have to be administered during a certain sequence of time during the day and when you say take it at 10:00, people will say what do you mean by 10:00?” [1].

Natsios was not the only policy maker to justify withholding ART from Africans on the basis that weak infrastructure, or patients' inability to take tablets, would stymie adherence. Senior officials of the World Bank and Thai government said in *The Lancet*: “[ART] is not…a technology that most poor people could adhere to…[Further] The use of public funds to subsidise the treatment of patients in the poorest countries who are most able to comply…would be highly inequitable” [2].

Two new systematic reviews prove these speculations were mistaken [3,4]. Despite their continent's poverty, and schooled or not in time keeping, Africans overcome these barriers and are better than North Americans at taking ART. These studies correct the misconception of earlier, nonsystematic reviews that concluded that Africans' adherence to medicines is “often poor” [5].

The first review (which I coauthored) identified 31 studies from North America and 27 from sub-Saharan Africa examining adherence to ART [3]. The bottom line was simple: using the customary definition that “good adherence” means taking ART as prescribed 95% of the time or more, then 82% of Africans succeeded at that goal, compared with only 55% of North Americans (*p* is less than 0.001).

Some may see this result as surprising. To live in Nairobi means to face so many privations compared to New York that to overcome them and excel seems almost storybook untrue. But privation can cut both ways. People who have been denied the necessities of life, who then receive the gift of medicines and a chance to live, may be more likely to appreciate ART.

Although Africans take ART more faithfully that North Americans, there is room for improvement. Here is where the second review is instructive [4]. The authors identified 84 studies from rich and poor countries that qualitatively or quantitatively identified factors impeding or facilitating adherence to ART. The impeding factors in rich and poor countries were familiar ones: patients' aversion or forgetfulness about medicines; lack of trust in health workers; fears about AIDS or its treatment; and emotions of isolation.

The authors found only two qualitative studies of barriers and facilitators of adherence among patients in poor countries [4]. There are accordingly few data on which to conclude that, for example, patients must give up alcohol, or must undergo directly observed therapy, to adhere to medicines, as some programs require [6,7]. Such measures may indeed be unnecessary.

In rich countries, the study failed to identify any obvious “big fix” that could turn non-adherent patients into adherent ones. On the other hand, for developing countries, “financial constraints” towered above the other reasons why poor patients may fail to adhere to ART. That is cruelly ironic, because the same international development policy makers who rejected the idea that poor people could adhere to ART also worked for financial donors such as USAID and the World Bank, and their passionate arguments against ART stalled the delivery of the one variable that helps adherence—money.

Where is the flaw that allowed speculation to get ahead of evidence in development policy making, and to reach the baseless conclusion that Africans could not adhere to ART, or needed to be commanded paternalistically (e.g., “you must abstain from alcohol”) to adhere to ART, when no such conclusion would be reached for rich people? More to the point, how can one recognize when a particular development policy is so baseless and speculative, the better to abandon it?

A serviceable answer, I believe, is that one should be highly suspicious whenever development policy makers sound dismissive of the people whom they are hired to help. The central aspiration of development work is helping the poor and sick become richer and healthier. Such an aspiration is incompatible with speculating that certain foreigners are incapable of enjoying the fruits of development. I believe that the views of Natsios and the World Bank and Thai officials, speculating that Africans could not adhere to ART, were dismissive in just this way.

Dismissing patients in this way leads to a lower standard of medical care. The medical establishment is more sensitive to the standard of care than is the development establishment, and so the medical establishment must be vigilant—and vocal—against bad development policy. Development policymakers have also freely opined that Africans could not manage to take artemisinin-based combination therapies for malaria, or second-line treatments for tuberculosis. We now know that Africans are capable of all these things—but overcoming the dismissals and excuses took years, during which millions died.
